# Peripartum administration of esketamine: a way to prevent postpartum depression?

**DOI:** 10.1192/j.eurpsy.2025.2402

**Published:** 2025-08-26

**Authors:** M. Del Valle Martín, R. De Hita Santillana, M. L. Costa Ferreira da Silva, C. Mouriño Sánchez

**Affiliations:** 1 Psychiatry, Hospital Universitario Severo Ochoa; 2 Psychiatry, Hospital Universitario José Germain, Leganes, Spain

## Abstract

**Introduction:**

Postpartum depression (PPD) is one of the most prevalent mental disorders, with the potential to precipitate complications in childbearing. The estimated prevalence of PPD ranges from 7% to 13% in developed countries and exceeds 20% in underdeveloped countries. Near 20% of women presenting with symptoms of PPD experience suicidal ideation. Prompt detection and management of this patient group is of particular importance. The use of esketamine has shown to reduce the incidence of PPD in previous studies, but its widespread use has been hampered by its side effects and the need for hospitalisation.

**Objectives:**

We aimed to review the available literature on the efficacy and safety of peripartum esketamine use.

**Methods:**

A narrative literature review was carried out in the PubMed, Cochrane and Embase databases, selecting only the articles published in the last 10 years, using the following keywords: pregnancy, esketamine, postpartum depression.

**Results:**

After childbirth 6 week is a high-risk period for PPD, specially the first week. The incidence of PPD is higher in C/S than in normal births. Compared with pregnant women who underwent C/S without the use of esketamine, those who used esketamine in the perioperative period had a 48% reduced risk of developing postpartum depression and a 1.43 point reduction in the Edinburgh Postnatal Depression Scale (EPDS). The optimal dose of esketamine for PPD is unknown; most studies consider high doses above 0.5 mg/kg and low doses inferior. These favourable effects were observed at both short and long-term follow-up and in low and high doses. Compared to intranasal esketamine, intravenous esketamine has a higher bioavailability and is more convenient to dose control. Route of administration and dose of esketamine did not affect the prophylactic effect of esketamine on PPD, but they differ in their adverse effect profiles. The incidence of immediate adverse reactions to intraoperative pumped esketamine is extremely high, particularly during the intraoperative period when more patients receiving esketamine developed neurological or psychiatric symptoms (97.7%). Other common immediate intraoperative maternal adverse events were nausea and vomiting, dizziness and hallucinations, but had no significant effect on postoperative adverse. Adverse reactions to esketamine are usually transient and are more common when single intravenous injection is used (continuous infusion is preferable), with faster and higher doses.

**Conclusions:**

PPD has potentially serious consequences for mothers and their children, and there is an urgent need for safe, effective and accessible treatments. As the use of esketamine has progressed, concerns have arisen about adverse effects, particularly long-term efficacy, addiction and suicide risk. Current evidence suggests that although it may have a good preventive effect, a long research trail is needed to prove and confirm the efficacy and safety of esketamine.

**Disclosure of Interest:**

None Declared

